# Neuronal Nitric Oxide Synthase as a Shared Target for the Effects of Adiponectin and Resistin on the Mechanical Responses of the Mouse Gastric Fundus

**DOI:** 10.3390/ijms232416113

**Published:** 2022-12-17

**Authors:** Eglantina Idrizaj, Silvia Nistri, Virginia Zizi, Maria Caterina Baccari

**Affiliations:** 1 Department of Experimental & Clinical Medicine, Section of Physiological Sciences, University of Florence, 50134 Florence, Italy; 2 Department of Experimental & Clinical Medicine, Research Unit of Histology & Embryology, University of Florence, 50139 Florence, Italy

**Keywords:** nitric oxide, resistin, adiponectin, gastric fundus, nNOS expression, adipokines, neuromodulation

## Abstract

It has been reported that adiponectin (ADPN) and resistin are co-secreted by white mouse adipocytes and exert similar inhibitory effects in the mouse gastric fundus, in which resistin was observed to increase neuronal nitric oxide synthase (nNOS) expression. On these grounds, the present work aimed to investigate whether the effects of the two adipokines on the neurally-induced relaxant responses potentiate each other and whether there is a possible correlation with changes in nNOS expression in preparations from the mouse gastric fundus. In carbachol (CCh)-precontracted strips, electrical field stimulation elicited nitrergic relaxant responses, whose amplitude was increased by ADPN or resistin, but no additional enhancements were observed in their concomitant presence. Western blot and immunofluorescence analyses revealed that ADPN, like resistin, was able to up-regulate nNOS expression and to increase the percentage of nNOS-positive neurons in the myenteric plexus: co-treatment with the two adipokines did not induce additional changes. The results indicate that the two adipokines modulate nitrergic neurotransmission, and both do so by up-regulating nNOS expression. Therefore, nNOS appears to be a shared target for the two adipokines’ effects, which, rather than mutually reinforcing each other, may represent a dual physiological control mechanism to guarantee gastric fundus relaxation.

## 1. Introduction

White adipose tissue is known to synthesize and release a variety of hormonal substances [1], named adipokines [2], which are able to act centrally or peripherally on different tissues and organs [3,4,5,6,7]. Adipokines have been reported to exert several actions in physiological and pathological conditions in both humans and animal models [8,9,10,11,12,13,14,15]. Among adipokines, adiponectin (ADPN) and resistin, which have been reported to reside within the same vesicles in mouse adipocytes and to be co-secreted in response to the same triggering signals [16], have been investigated for their roles in both physiological and pathophysiological conditions [6,17,18]. ADPN and resistin, with their different actions, have been reported to also be able to affect gastrointestinal functions [19], including motility [20,21]. In this view, it has been previously observed that in strips from the mouse gastric fundus, ADPN and resistin exert inhibitory effects that appeared to occur, at least in part, through a nitric oxide (NO)-dependent mechanism [21,22,23,24]. Indeed, gastric motility is known to be under hormonal and nervous control. The latter is represented, other than by excitatory nervous fibers (mainly cholinergic), by non-adrenergic, non-cholinergic (NANC) fibers. NO is considered the main inhibitory neurotransmitter released by NANC fibers supplying the gastric smooth muscle and is responsible for relaxation in both humans and rodents [25,26,27]. NO synthesis is known to occur from L-arginine under the catalytic action of different NO synthase (NOS) isoforms. Endothelial NOS (e-NOS or NOS III), neuronal NOS (nNOS or NOS I) and inducible NOS (iNOS or NOS II) represent the three major isoforms, usually expressed by different cell types [28]. In particular, nNOS appears to be the main isoform able to produce NO as a neurotransmitter, which indeed plays the most important role in the control of gastrointestinal motility [25,29]. In this view, a significant increase in nNOS expression in neurons of the myenteric plexus following resistin exposure has been revealed in gastric specimens from mice [24].

Based on the observation that ADPN and resistin exert similar inhibitory effects on the mouse gastric fundus, at least in part through a NO-dependent mechanism, along with the finding that the two adipokines are co-secreted by white mouse adipocytes [16], the present work aimed to investigate whether their effects on neurally-induced inhibitory responses mutually enhance each other at the level of the stomach and whether the nNOS isoform is involved in the effects of both hormones. For this purpose, the combined action of the two adipokines on either the neurally-induced relaxant responses or nNOS expression was evaluated in preparations from the mouse gastric fundus.

## 2. Results

### 2.1. Functional Experiments

**Figure 1:**
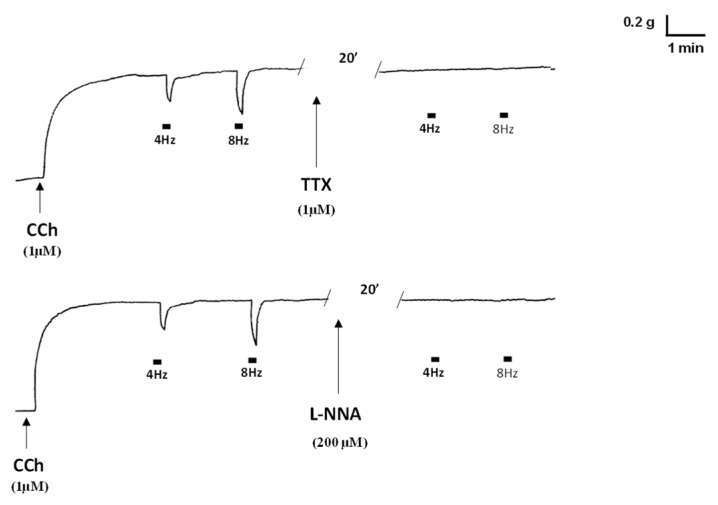
Effects of TTX and L-NNA on the EFS-induced inhibitory responses in CCh-precontracted gastric strips. Typical tracing showing the relaxant responses obtained at 4 and 8 Hz stimulation frequencies in carbachol (CCh)-precontracted strips and in the presence of 1µM guanethidine (upper and lower left-hand traces) in strips from the mouse gastric fundus. Note the abolition of the electrical field stimulation (EFS)-induced inhibitory responses at both stimulation frequencies following the addition of tetrodotoxin (TTX) (upper right-hand trace) or the nitric oxide (NO) synthesis inhibitor L-N^G^-nitro arginine (L-NNA) (lower right-hand trace) to the bath medium.

As previously observed [23,24], in strips from the mouse gastric fundus, the addition of 1µM carbachol (CCh) to the bath medium (*n* = 20) caused a rapid rise in contraction (mean amplitude 1.1 ± 0.2 g) (Figure 1), which persisted until washout. In CCh-precontracted strips, electrical field stimulation (EFS, 4 and 8 Hz) elicited a fast relaxant response, which persisted throughout the whole period of stimulation. At the end of the stimulation period, the strip tension regained its baseline value (Figure 1). The addition of either 1µM tetrodotoxin (TTX) (*n* = 3) or 200µM L-N*^G^*-nitro arginine (L-NNA) (*n* = 3), a NO synthesis inhibitor, to the bath medium caused the abolition of the EFS-evoked relaxant responses, indicating their nervous and nitrergic nature, respectively (Figure 1).

**Figure 2:**
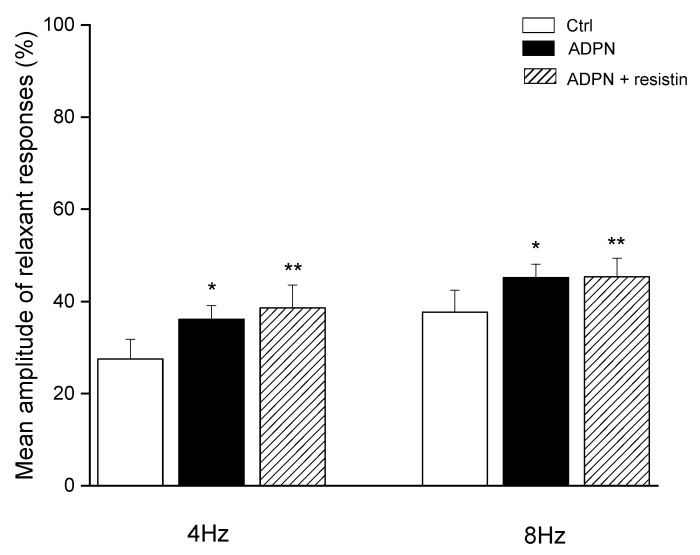
Mean amplitude of the EFS-induced relaxant responses in the presence of adiponectin (ADPN) and following the subsequent addition of resistin to the bath medium. Bar chart showing the significant increase in the mean amplitude of electrical field stimulation (EFS)-evoked relaxant responses at 4 and 8 Hz stimulation frequencies evoked by adiponectin (ADPN) (20 nM) and the lack of additional effects of resistin (60 ng/mL) added to the bath medium 30 min after ADPN. Amplitude values refer to the maximum peaks obtained during the stimulation period and represent percentage decreases relative to the muscular tension induced by carbachol (CCh, 1µM), taken as 100%. All values are means ± SEM of 4 strips. * *p* < 0.05 vs. its own control (Ctrl); ** *p* < 0.05 vs. its own Ctrl and *p* > 0.05 vs. ADPN (one-way ANOVA followed by Newman–Keuls post-test).

The addition of ADPN (20 nM) to the bath medium (*n* = 4) caused a statistically significant increase in the mean amplitude of EFS-evoked relaxation (*p* < 0.05) at both stimulation frequencies employed (Figure 2). The effects of the hormone were already detectable 15–20 min after its inclusion in the bath medium. Thirty minutes after the addition of 20 nM ADPN to the bath medium, the inclusion of resistin (60 ng/mL) (*n* = 4) did not cause any further significant effects (*p* > 0.05) on the amplitude of the relaxant responses at either stimulation frequency employed (Figure 2).

**Figure 3:**
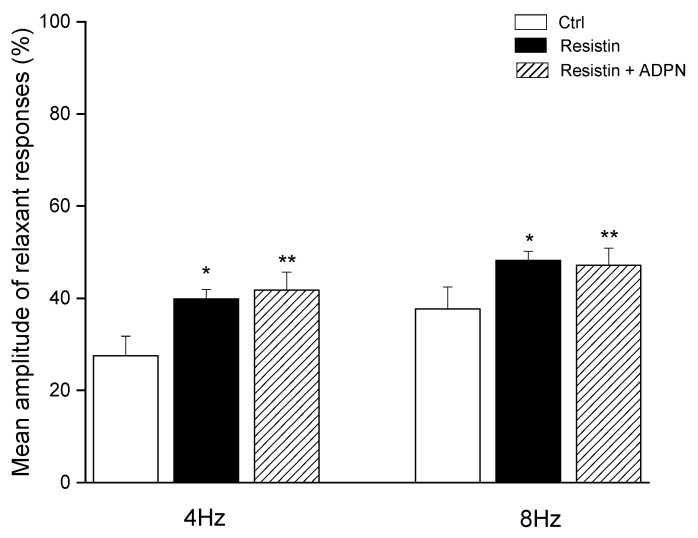
Mean amplitude of the EFS-induced relaxant responses in the presence of resistin and following the subsequent addition of adiponectin (ADPN) to the bath medium. Bar chart showing the significant increase in the mean amplitude of the electrical field stimulation (EFS)-induced relaxant responses at 4 and 8 Hz stimulation frequency evoked by resistin (60 ng/mL) and the lack of additional effects of adiponectin (ADPN, 20 nM) added to the bath medium 30 min after resistin. Amplitude values refer to the maximum peaks obtained during the stimulation period and represent percentage decreases relative to the muscular tension induced by carbachol (CCh, 1 µM), taken as 100%. All values are means ± SEM of 4 strips. * *p* < 0.05 vs. its own control (Ctrl); ** *p* < 0.05 vs. its own Ctrl and *p* > 0.05 vs. resistin (one-way ANOVA followed by Newman–Keuls post-test).

**Figure 4:**
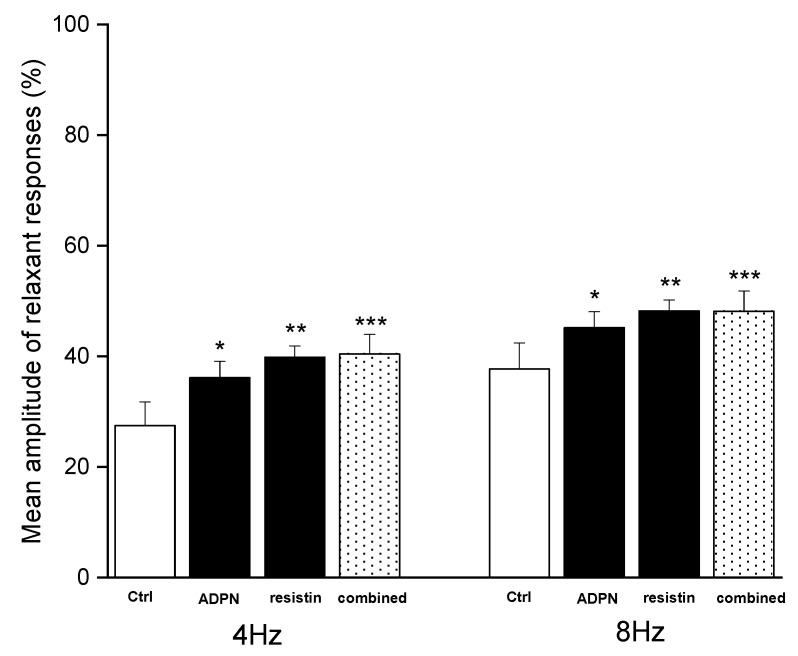
Lack of additional effects on the mean amplitude of the EFS-induced relaxant responses after the simultaneous inclusion of the two adipokines to the bath medium. Bar chart showing the significant increase in the mean amplitude of electrical field stimulation (EFS)-evoked relaxant responses at 4 and 8 Hz stimulation frequency evoked by adiponectin (ADPN, 20 nM), resistin (60 ng/mL) or the simultaneous inclusion of the two adipokines to the bath medium. Note the apparently more pronounced influence, although not statistically different, of resistin compared to ADPN and the lack of additional effects of the two adipokines when simultaneously added to the bath medium. Amplitude values refer to the maximum peaks obtained during the stimulation period and represent percentage decreases relative to the muscular tension induced by carbachol (CCh, 1µM), taken as 100%. All values are means ± SEM of 4/6 strips. * *p* < 0.05 vs. its own control (Ctrl); ** *p* < 0.05 vs. its own Ctrl and *p* > 0.05 vs. ADPN; *** *p* < 0.05 vs. its own Ctrl and *p* > 0.05 vs. ADPN or resistin (one-way ANOVA followed by Newman–Keuls post-test).

Resistin (60 ng/mL) caused (*n* = 4) a statistically significant increase in the mean amplitude of EFS-evoked relaxation (*p* < 0.05) at both stimulation frequencies employed (Figure 3). The effects of the hormone were already detectable 15–20 min after its inclusion in the bath medium. The increase in the amplitude of the neurally-induced relaxant responses caused by resistin appeared more pronounced, although not statistically different (*p* > 0.05), compared to that evoked by ADPN at both stimulation frequencies employed (Figure 4). Thirty minutes after the addition of 60 ng/mL resistin to the bath medium, the inclusion of ADPN (20 nM) (*n* = 4) did not cause any further significant increases (*p* > 0.05) in the amplitude of the relaxant responses at either stimulation frequency employed (Figure 3).

To confirm the above observations, the effects on the EFS-induced relaxant responses caused by the simultaneous addition of both adipokines to the bath medium (*n* = 6) were also tested. In these conditions, the mean amplitude of the EFS-induced relaxant responses was significantly increased (*p* < 0.05) (Figure 4). However, this increase in amplitude was not statistically different (*p* > 0.05) from that observed when the hormones were individually added to the bath medium (Figure 3). The effects of the hormones were already detectable 15–20 min after their inclusion in the bath medium and persisted for up to 1 h (no longer time observed).

### 2.2. Western Blotting and Immunofluorescence Analysis

**Figure 5:**
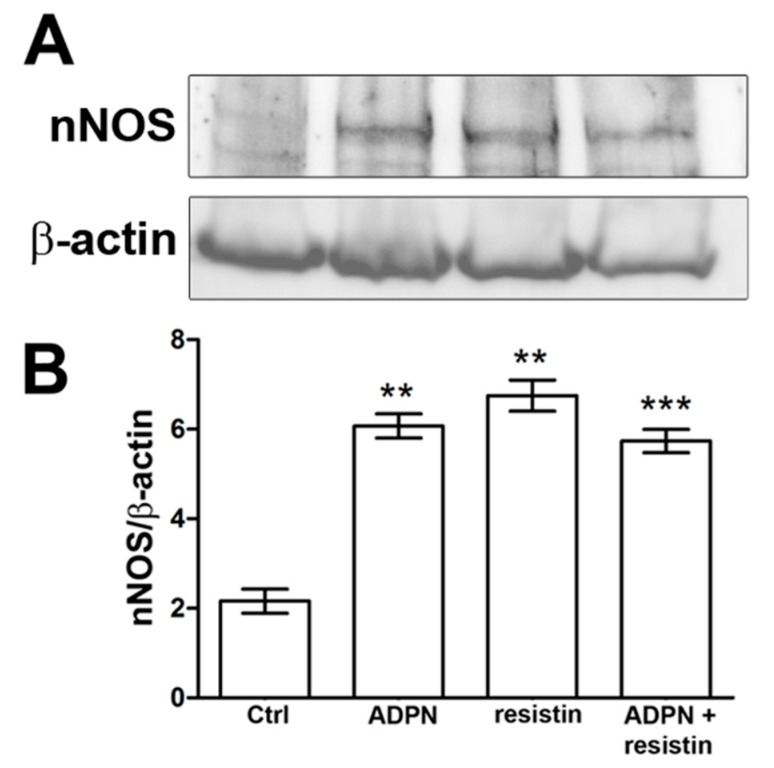
Effects of the different hormones on nNOS expression in the mouse gastric fundus assayed by Western blotting. (**A**) Representative bands from a typical experiment. (**B**) Quantitative analysis. Significance of differences (one-way ANOVA and Newman–Keuls post-test for multiple comparisons): ** *p* < 0.01 vs. controls (Ctrl) and *p* > 0.05 vs. adiponectin (ADPN) or resistin; *** *p* < 0.001 vs. Ctrl and *p* > 0.05 vs. ADPN or resistin. The reported data are expressed as the means ± SEM of 3 samples for each experimental group.

**Figure 6:**
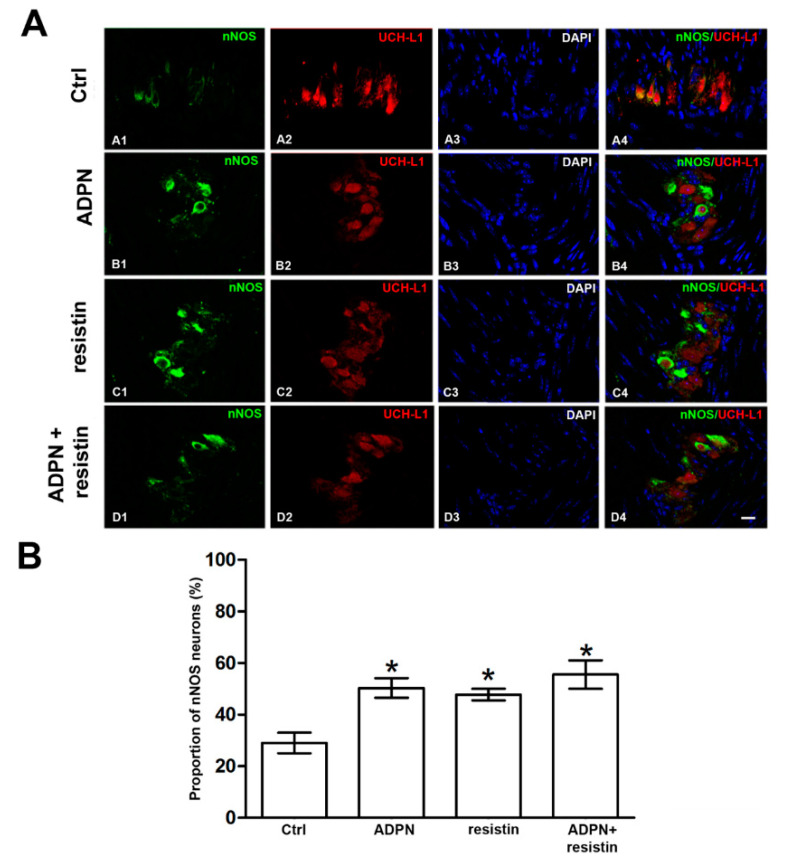
Effects of the different hormones on nNOS-positive neurons in the mouse gastric fundus assayed by immunofluorescence analysis. (**A**) Representative photomicrographs of gastric tissue from control and hormone-exposed samples showing double immunofluorescence labeling. (A1–A4) Co-localization of neuronal nitric oxide synthase (nNOS) and ubiquitin carboxy-terminal hydrolase-L1 (UCH-L1) in control mice: (A1) nNOS signal (green channel); (A2) UCH-L1 signal (red channel); (A3) 4′,6-diamidino-2-phenylindole (DAPI, blue channel); (A4) nNOS/UCH-L1 signals (merged images). (B1–B4) Co-localization of nNOS and UCH-L1 in ADPN-exposed preparations: (B1) nNOS signal (green channel); (B2) UCH-L1 signal (red channel); (B3) DAPI (blue channel); (B4) nNOS/UCH-L1 signals (merged images). (C1–C4) Co-localization of nNOS and UCH-L1 in resistin-exposed preparations: (C1) nNOS signal (green channel); (C2) UCH-L1 signal (red channel); (C3) DAPI (blue channel); (C4) nNOS/UCH-L1 signals (merged images). (D1–D4) Co-localization of nNOS and UCH-L1 in ADPN and resistin-exposed preparations: (D1) nNOS signal (green channel); (D2) UCH-L1 signal (red channel); (D3) DAPI (blue channel); (D4) nNOS/UCH-L1 signals (merged images). Scale bar: 20 μm. (**B**) Quantitative analysis of the percentages of nNOS-positive neurons present in the myenteric plexus from the specimens exposed to the different treatments. Columns are means ± SEM of 4–6 samples for each experimental group. Significance of differences (one-way ANOVA and Newman–Keuls post-test for multiple comparisons): * *p* < 0.05 vs. controls (Ctrl) and *p* > 0.05 vs. adiponectin (ADPN) or resistin.

The exposure of gastric fundus specimens to ADPN (20 nM) or resistin (60 ng/mL) induced a significant increase in nNOS expression compared to the controls (Figure 5). However, the effects of the two adipokines on nNOS expression were not statistically different to each other (Figure 5). Co-treatment with ADPN (20 nM) and resistin (60 ng/mL) did not cause any additional increases in nNOS expression (Figure 5). Immunofluorescence analysis confirmed the data obtained by Western blotting. In specimens exposed to ADPN (20 nM), resistin (60 ng/mL) or both hormones, the number of nNOS-positive neurons significantly increased compared to the controls, but no differences were found among the hormone-exposed groups (Figure 6).

## 3. Discussion

The present results indicate that ADPN and resistin exert a modulatory role on nitrergic neurotransmission, and both do so by up-regulating the expression of nNOS, which appears to be a shared target for their effects on the neurally-induced relaxant responses in strips from the mouse gastric fundus.

In this view, ADPN and resistin have been reported to affect gastric activity in rodents by exerting inhibitory effects, thereby facilitating smooth muscle relaxation [21,22,23,24,30].

In the present experiments, EFS-induced relaxant responses were abolished by TTX or the NO synthesis inhibitor L-NNA, indicating their nervous and nitrergic nature. Therefore, the ability of ADPN or resistin to increase the amplitude of the neurally-induced relaxant responses supports the notion that both adipokines act by modulating nitrergic neurotransmission in preparations from the mouse gastric fundus, in keeping with previous reports [23,24].

In the current work, the observation that, in the presence of ADPN, the subsequent inclusion of resistin in the bath medium did not cause any additional effects on the amplitude of the neurally-induced relaxant responses, and vice versa, indicates that the two adipokines exert their effects by engaging the same mechanism. In agreement, Western blot and immunofluorescence analyses showed an increase in the expression of the nNOS isoform and in the number of nNOS-positive neurons in specimens following their exposure to ADPN, similar to what was previously observed with resistin in preparations from the mouse gastric fundus [24].

Moreover, the lack of additional effects on the amplitude of EFS-evoked relaxation when the two adipokines were simultaneously included in the bath medium, though supporting the engagement of the same mechanism by the two adipokines, was, at the same time, quite a surprising result. Given the similar action exerted by the two adipokines at the gastric fundus level and the ability of the mouse adipose tissue to co-release the two adipokines [16], it was plausible to expect that the effects of ADPN and resistin might actually serve to strengthen each other.

On the other hand, in the present experiments, Western blot and immunofluorescence analyses revealed no differences in nNOS expression or in the number of nNOS-positive neurons among specimens exposed to ADPN or resistin or even to their concomitant presence.

Therefore, taken together, data from the present multidisciplinary study suggest that ADPN and resistin affect the neurally-induced relaxant responses through a modulatory role on the nitrergic neurotransmission, and both do so by up-regulating the expression of nNOS, which appears to be a shared target for the effects of the two adipokines in the mouse gastric fundus. Indeed, nNOS has been reported as the main isoform in the enteric nervous system able to produce NO as an inhibitory NANC neurotransmitter, which plays the most important role in the control of gastrointestinal motility [25,29]. In this view, the nNOS isoform has been reported as a shared target in the control of gastrointestinal motility by several hormones [25,31] to modulate the amount of NO production. Changes in the expression of different NOS isoforms have indeed been revealed in both physiological and pathophysiological conditions, particularly those related to the nNOS isoform in the gut enteric nervous system [32,33]. Reduced nNOS expression in enteric inhibitory motor neurons indeed appears to be involved in gut dysmotility, and NO overproduction in some inflammatory conditions could impair motor activity in different portions of the gastrointestinal tract of both humans and animals [29,33,34]. Therefore, the mechanisms that regulate NO production seem essential for the maintenance of physiological NO concentrations to preserve its biological functions or to control its harmful effects. Thus, the action of both adipokines on nNOS expression, without potentiating each other, could be speculated as a mechanism for avoiding excessive NO production and, at the same time, for ensuring its physiological effects. This is of particular importance in the control of the motor responses of the proximal stomach, whose main physiological functions involve NO. NO has indeed been reported to cause gastric fundus relaxation in both humans and animals and to be involved in the maintenance of the basal tone as well as in the accommodation of the proximal stomach [34,35]. In this view, defects in nNOS-positive neurons or nitrergic neurotransmission have been reported to be responsible for gastric motility disorders [34,36].

In conclusion, the present results indicate that the two adipokines modulate nitrergic neurotransmission, and both do so by up-regulating nNOS expression and increasing the percentage of nNOS-positive neurons in the myenteric plexus of the mouse gastric fundus. Therefore, nNOS appears to be a shared target for the two hormones’ effects, which, rather than mutually reinforcing each other, may represent a dual physiological control mechanism aimed at ensuring gastric fundus relaxation, further underlying its importance.

## 4. Materials and Methods

### 4.1. Animals and Ethical Approval

Experiments were conducted on female mice (C57BL/6J; ENVIGO, Udine, Italy) aged 8 to 12 weeks old. The animals, fed standard laboratory chow and water, were housed at a controlled temperature (21 ± 1 °C) and under a 12 h light/12 h dark photoperiod. The experimental protocol was designed in accordance with the guidelines of the European Communities Council Directive 2010/63/UE and the recommendations for the care and use of laboratory animals approved by the Animal Care Committee (University of Florence, Florence, Italy), subject to the authorization of the Italian Ministry of Health (code 0DD9B.N.ZB6/2020 to MCB). The animals were sacrificed by cervical dislocation to minimize animal suffering.

### 4.2. Mechanical Recording

As previously reported [23], the stomach was quickly removed from the abdomen of ten mice, and two full-thickness strips (2 × 10 mm) were cut in the direction of the longitudinal muscle layer from the fundus region. One end of each strip was tied to a platinum rod, while the other was connected to a force displacement transducer (Grass model FT03, Quincy, MA, USA) by a silk thread for the continuous recording of isometric tension. The transducer was coupled to polygraph systems (Grass model 7K, Quincy, MA, USA). Preparations were mounted in the longitudinal direction in 5 mL double-jacketed organ baths containing Krebs–Henseleit solution, gassed with a 95% O_2_−5% CO_2_ mixture, of the following composition (mM): NaCl 118, KCl 4.7, MgSO_4_ 1.2, KH_2_PO_4_ 1.2, NaHCO_3_ 25, CaCl_2_ 2.5 and glucose 10 (pH 7.4). Prewarmed water (37 °C) circulated through the outer jacket of the tissue bath via a constant-temperature circulator pump. The temperature of the Krebs–Henseleit solution in the organ bath was maintained within ±0.5 °C.

Electrical field stimulation (EFS) was applied via two platinum wire rings (2 mm diameter, 5 mm apart) through which the preparation was threaded. Electrical pulses (rectangular waves, 80 V, 4 and 8 Hz, 0.5 ms, for 15 s) were provided by a Grass model S8 stimulator. Strips were allowed to equilibrate for 1 h under an initial load of 0.8 g. During this period, repeated and prolonged washes of the preparations with Krebs–Henseleit solution were performed to prevent the accumulation of metabolites in the organ baths.

All functional experiments were performed in the presence of carbachol (CCh, 1 µM) and guanethidine (1 µM) to prevent cholinergic and adrenergic influences, respectively. When contraction elicited by CCh reached a stable plateau phase, EFS or drugs were applied. The interval between two subsequent applications of CCh was no less than 15 min, during which repeated and prolonged washes with Krebs–Henseleit solution were performed.

In the first series of experiments, the effects of tetrodotoxin (TTX, 1 µM) or the NO synthesis inhibitor L-NG-nitro arginine (L-NNA, 200 µM) on the EFS-induced inhibitory responses at 4 and 8 Hz stimulation frequencies were tested for each treatment in 3 gastric fundus strips.

In the second series of experiments, the influence of adiponectin (ADPN, 20 nM) on the EFS-induced relaxant responses was tested in 4 strips. Thirty minutes after, when the effects of ADPN were fully manifested, resistin (60 ng/mL) was included in the bath medium, and EFS was applied again for at least another 30–40 min.

In the third series of experiments, the influence of resistin (60 ng/mL) on the EFS-evoked relaxant responses was tested in 4 strips. Thirty minutes after, when the effects of resistin were fully manifested, ADPN (20 nM) was included in the bath medium, and EFS was applied again for at least another 30–40 min.

Another series of experiments were then performed, in which the effects of the two adipokines on the EFS-induced relaxant responses were investigated in 6 strips when ADPN (20 nM) and resistin (60 ng/mL) were simultaneously added to the bath medium.

### 4.3. Western Blotting and Immunofluorescence Analysis

Gastric fundus specimens were taken and stabilized in 5 mL organ baths containing Krebs–Henseleit solution. At the end of the stabilizing period, one-half of the specimens were exposed for 30 min to resistin (60 ng/mL), ADPN (20 nM) or both hormones simultaneously. The second half was maintained in Krebs solution for the same amount of time without hormone addition (controls), and then preparations were immediately processed for Western blot and immunofluorescence analyses.

#### 4.3.1. Western Blotting

Fragments of gastric fundi from the control and hormone-exposed specimens (3 samples for each experimental group) were homogenized in cold lysis buffer (20 mmol /L Tris/HCl (pH 7.4), 10 mmol/L NaCl, 1.5 mmol/L MgCl2, 5 mmol/L EGTA, 2 mmol/L Na2EDTA, mixed with 10× Sigmafast Protease Inhibitor Cocktail tablets). The total protein content was measured spectrophotometrically using the micro-BCA™ Protein Assay Kit (Pierce, IL, USA). Fifty micrograms of total protein was electrophoresed by SDS–PAGE and blotted onto PVDF membranes (Millipore, Bedford, MA, USA). The membranes were incubated overnight (O.N.) at 4 °C with rabbit polyclonal anti-nNOS (1:2000; Millipore) and rabbit polyclonal anti-β-actin (1:20,000; Sigma Aldrich, St. Louis, MO, USA), assuming β-actin as a control invariant protein. Specific bands were detected using rabbit peroxidase-labeled secondary antibodies (1:15,000 Vector, Burlingame, CA, USA) and enhanced chemiluminescent substrate. Densitometric analysis of the bands was performed using Scion Image Beta 4.0.2 image analysis software (Scion Corp., Frederick, MD, USA).

#### 4.3.2. Immunofluorescence Analysis

Gastric tissue samples were fixed in 4% paraformaldehyde, embedded in paraffin and cut into 5 µm thick sections. For antigen retrieval, the sections were deparaffinized, rehydrated and maintained in EDTA 1 mmol/L, pH 9.0, + tris buffer 10 mmol/L, for 20 min at a temperature of 90–92 °C [24]. To quench the autofluorescence of elastic fibers, the sections were incubated in 2 mg/mL glycine (AppliChem, Darmstadt, Germany) for 8 min at room temperature (RT). To minimize unspecific binding, the sections were preincubated with 1.5% bovine serum albumin (Sigma Aldrich) for 20 min at RT and then incubated O.N. at 4 °C with rabbit monoclonal anti-nNOS antibody (1:2000, Millipore, Bedford, MA, USA) followed by goat anti-rabbit Alexa Fluor 488-conjugated IgG (1:350, Invitrogen, San Diego, CA, USA) for 2 h at RT. After the first incubation described above, the sections were reincubated O.N. at 4 °C with anti-mouse monoclonal anti-ubiquitin carboxy-terminal hydrolase-L1 (UCH-L1), a neuronal marker antibody (1:200; Santa Cruz Biotechnology, Texas, USA), and then incubated with the appropriate Alexa Fluor 568-conjugated IgG (1:350; Invitrogen) for 2 h at RT. Negative controls were performed by omitting the primary antibodies. The sections were mounted with FluoroshiedTM mounting medium containing the nuclear marker 4′,6-diamidino-2-phenylindole (DAPI, Sigma Aldrich) and observed under a confocal Leica Stellaris 5 microscope (Leica Microsystems, Mannheim, Germany) equipped with a White-Light Laser (WLL) source for fluorescence measurements and coupled to LAS X microscope software (Leica). Observations were performed using a Leica HC PL Apo 20X/0.75 CS2 objective.

The number of nNOS neurons was evaluated within myenteric ganglia along the entire section (4–6 samples for each experimental group) by two observers who were blinded to each other, and the results are expressed as the percentage of nNOS-positive neurons to total UCH-L1 neurons.

### 4.4. Drugs

Guanethidine sulfate, carbachol (CCh), mouse recombinant resistin, recombinant full-length mouse adiponectin (ADPN), tetrodotoxin (TTX) and L-N^G^-nitro arginine (L-NNA) were obtained from Sigma Chemical (St. Louis, MO, USA), while resistin was purchased from PeproTech (London, UK). Solutions were prepared on the day of the experiment, except for TTX, ADPN and resistin, for which stock solutions were kept stored at −20 °C. Drug concentrations are referred to as final bath concentrations and are in the range of those previously reported to be effective in isolated smooth muscle tissues [23,24,37,38].

### 4.5. Data Analysis and Statistical Tests

Relaxant responses are expressed as a percentage decrease relative to the muscular tension induced by 1 × 10^−6^ M CCh just before obtaining relaxations and measured 30 s after a stable plateau phase was reached. Amplitude values of EFS-induced relaxations refer to the maximum peaks obtained during the stimulation period.

Calculations were performed using GraphPad Prism 2.0 statistical program (GraphPad Software, San Diego, CA, USA). The statistical significance was assessed by one-way ANOVA followed by Newman–Keuls post-test for multiple comparisons. Differences were considered significant when *p* < 0.05. Results are means ± SEM. The number of preparations is indicated by *n* in the results.

## Data Availability

Not applicable.

## Abbreviations

ADPN	Adiponectin
CCh	Carbachol
CTRL	Control
EFS	Electrical field stimulation
L-NNA	L-N^G^-nitro arginine
NANC	Non-adrenergic, non-cholinergic
NO	Nitric oxide
NOS	Nitric oxide synthase
nNOS	Neuronal nitric oxide synthase
TTX	Tetrodotoxin
